# Hirschsprung's disease prognosis: significance of the length of aganglionosis and reference value for the dilated segment resection length

**DOI:** 10.3389/fped.2025.1553317

**Published:** 2025-06-16

**Authors:** Yingyu Jia, Bingliang Li, Hongwei Xi, Hongxia Ren

**Affiliations:** ^1^ Neonatal Surgery, Shanxi Children’s Hospital, Taiyuan, China; ^2^ Department of Pediatrics, Shanxi Medical University, Taiyuan, China; ^3^ General Surgery, Shanxi Children’s Hospital, Taiyuan, China

**Keywords:** Hirschsprung's disease, dilated segment resection length, short-term clinical outcome, receiver operating characteristic (ROC) curve, the length of aganglionosis

## Abstract

**Background:**

The appropriate length of resection for the dilated segment in Hirschsprung's disease (HSCR) remains a subject of debate, and the correlation between postoperative clinical outcomes has yet to be elucidated. This study aimed to explore the relationship between the dilated segment resection length (DSRL) and the short-term clinical outcome of HSCR, as well as to determine the optimal DSRL value.

**Methods:**

The clinical data of all children with HSCR who underwent a pull-through surgery at Shanxi Children's Hospital from May 2016 to September 2023 were analyzed retrospectively, the baseline characteristics such as sex, gestational age, family history, and complications such as soiling, perianal erosion, constipation were collected. The groups were stratified in recto-sigmoid aganglionosis (short-segment) and extended colonic (long-segment), and DSRL was divided into three groups: DSRL < 10 cm, 10 ≤ DSRL < 20 cm, and DSRL ≥ 20 cm. The Wingspread score system was used to evaluate anal function and analyze the short-term clinical outcome.

**Results:**

A total of 223 children were included in the study, among which 104 cases had short-segment HSCR and 119 cases had long-segment HSCR. The median age at which pull-through surgery was performed was 4 months. In cases of short-segment HSCR, aside from preoperative anemia, baseline characteristics showed no statistically significant differences among the three groups. No statistically significant association was observed between DSRL, the total length of intestinal resection, the length of aganglionosis,and postoperative clinical outcomes.For short-segment HSCR, the best postoperative bowel function was observed when DSRL < 10 cm, with the optimal value being 7.25 cm. In cases of long-segment HSCR, no statistically significant differences in baseline characteristics were observed among the three groups. DSRL, the total length of intestinal resection and the length of aganglionosis all showed statistically significant differences in relation to soiling and perianal erosion. For long-segment HSCR, the best postoperative bowel function was observed when 10 ≤ DSRL < 20 cm, with the optimal value being 13.00 cm.

**Conclusions:**

Not only the dilated segment resection length matters for the outcome but also the length of aganglionosis. For short-segment HSCR, DSRL, the total length of intestinal resection and the length of aganglionosis showed no significant impact on short-term clinical outcomes. In contrast, these parameters in long-segment HSCR were significantly associated with soiling and perianal erosion, although overall patient quality of life remained satisfactory. Data from a single clinical center suggest that optimal clinical outcomes for children are achieved when the DSRL measurements are 7.25 cm for short-segment HSCR and 13.00 cm for long-segment HSCR.

## 1 Introduction

Hirschsprung's disease (HSCR) is a congenital intestinal disease resulting from the incomplete migration of enteric neural crest cells during embryonic development (1). Histologically, it is defined by the absence of enteric ganglion cells in the submucosal and myenteric plexuses (2). HSCR is mainly treated by transanal endorectal pull-through (TEPT), and most patients have satisfactory postoperative outcomes and good long-term prognosis, but some patients still have intestinal dysfunction, such as soiling, constipation, and Hirschsprung's disease associated enterocolitis (HAEC) (3). The association between these complications and the length of the dilated segment resection (DSRL) remains controversial in current literature.

Many scholars argue that the DSRL should be greater than 5 cm (4, 5). However, Ullrich et al. (6) Found that DSRL < 5 cm did not affect the occurrence of postoperative complications. Insufficient resection of the dilated segment may result in residue of the transition zone (TZ). Conversely, excessive resection length can extend the duration of the surgical procedure and elevate intraoperative blood loss, in addition to adversely influencing the colon's ability to absorb water (7), which may subsequently affect growth and development. Therefore, the determination of optimal DSRL remains an academic issue that requires in-depth exploration.

Given the existing discrepancies in DSRL recommendations and the paucity of subtype-specific studies in HSCR, further investigation into the relationship between DSRL and postoperative clinical outcomes is of significant importance. This study aimed to analyze single-center clinical data by categorizing patients into short-segment and long-segment groups based on the extent of aganglionosis. It explores the impact of DSRL on short-term postoperative clinical outcomes in different subgroups and provides evidence-based recommendations for the optimal DSRL value.

## 2 Methods

### 2.1 Settings and data collected

**Figure 1:**
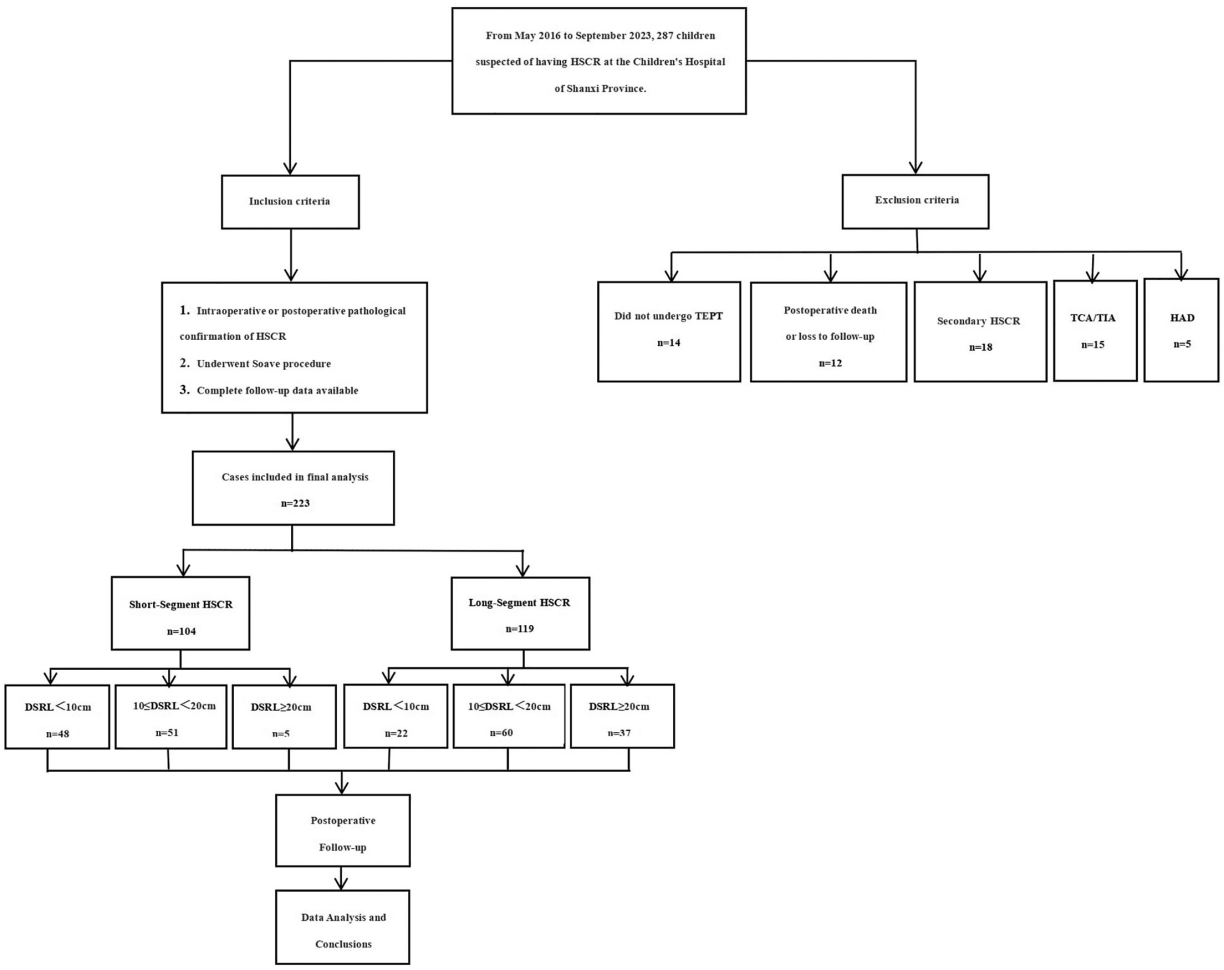
Study flow diagram.

After approved and supervised by the Ethics Committee of Shanxi Children's Hospital (IRB-WZ-2024-020), a retrospective analysis was conducted on all children with HSCR who received TEPT in our hospital from May 2016 to September 2023. Diagnosis was confirmed by histological examination. The resected specimens were evaluated for ganglion cells using multiplanar full-thickness biopsies combined with calretinin immunohistochemical staining, with independent confirmation by two senior pathologists.The operative age, sex, gestational age, family history, parity, preoperative enterocolitis, pre pull-through ostomy, hypoalbuminemia, anemia, DSRL, the total length of intestinal resection, pathological classification, surgical method, and postoperative complication were collected. The groups were stratified in recto-sigmoid aganglionosis (short-segment) and extended colonic (long-segment), and DSRL was divided into three groups: DSRL < 10 cm, 10 ≤ DSRL < 20 cm, DSRL ≥ 20 cm. The clinical outcomes such as constipation, HAEC, soiling, perianal erosion, reoperation, and medical therapy were followed up at 1, 3, and 6 months after operation. Wingspread score system was used to evaluate the bowel function and calculate the excellent and good rates (8). Excellent and good rate = (number of people judged as “excellent” + number of people judged as “good”)/total number of cases × 100%. In this study, the follow-up was completed by the same medical staff. The follow-up scoring was calculated based on standardized criteria and the type of symptoms (episodes of incontinence, stool consistency, and frequency) (Figure 1).

To ensure data integrity and cohort homogeneity, we excluded patients who did not undergo TEPT, those with postoperative death or loss of follow-up, secondary HSCR, total colonic HSCR or total intestinal HSCR(TCA/TIA), and intraoperative pathological diagnosis of Hirschsprung's disease allied diseases (HAD)(including intestinal neuronal dysplasia, isolated hypoganglionosis, immaturity of ganglia and so on) (9). All patients underwent Soave procedure, including transanal or laparoscopically assisted Soave procedure.

### 2.2 Surgical techniques

Transanal Soave Procedure: Following anesthesia induction, patients were positioned in lithotomy position. After standard disinfection and draping, a urinary catheter was inserted, and an anal retractor was placed. A circumferential incision was made 0.5–1.0 cm above the dentate line. The rectal mucosa was dissected proximally along the submucosal plane into the abdominal cavity. Partial resection of the posterior rectal muscular cuff was performed. After ligation of mesenteric vessels, the aganglionic bowel was exteriorized through the anal canal. Intraoperative frozen section examination was performed at the proximal resection margin to confirm the presence of ganglion cells. The affected bowel segment was then resected, followed by a single-layer anastomosis between the pulled-through colon and the residual rectal stump above the dentate line (10).

Laparoscopic-assisted Soave Procedure: After anesthesia induction, patients were positioned supine. Following standard disinfection and draping, a urinary catheter was inserted. A 5-mm trocar was placed through an umbilical incision to establish pneumoperitoneum, with additional 2–3 trocars inserted through the abdominal wall. The aganglionic segment was identified and mobilized using laparoscopic techniques, with mesenteric vessels divided using a harmonic scalpel. The patient was then repositioned to lithotomy position, and the subsequent steps were identical to the transanal Soave procedure as described above (11).

### 2.3 Definitions

Short-segment HSCR is defined as aganglionosis confined to the rectosigmoid region (not extending proximal to the sigmoid colon), while long-segment HSCR is defined as aganglionosis extending beyond the sigmoid colon, involving the descending/transverse/ascending colon (extensive aganglionosis). The transitional zone (TZ) is defined as the length of colon between the part in which the ganglion cells are there, but not sufficient, and the part where there are a normal amount of ganglion cells. The length from the proximal end of the TZ to the intestinal stump is the DSRL. Secondary HSCR is defined as acquired aganglionosis or dysfunction of enteric ganglia caused by other definitive etiologies (e.g., infection, ischemia, obstruction, etc.). While its clinical manifestations resemble those of HSCR, the underlying pathogenesis and pathological basis are distinct. Constipation was diagnosed according to the Rome IV standard (12), and soiling is defined as the involuntary leakage of a small amount of stool or stool-like liquid by the child, necessitating a change of diaper or underwear. HAEC was defined as occurring ≥2 times and diagnosed based on the Pastor scoring system (≥10 points) (13).

### 2.4 Statistical analysis

Classification variables are represented as counts and percentages, while continuous variables are represented as median and interquartile range (IQR). In our sample, the normality of the data was assessed using the Kolmogorov–Smirnov test. For continuous variables that did not follow a normal distribution, the Kruskal–Wallis test was employed for intergroup comparisons. For categorical variables, the chi-square test was utilized for analysis. Continuous variables that adhered to a normal distribution were expressed as $\bar{X}$ ±S, and the Pearson correlation coefficient was applied to evaluate the relationships between variables. The receiver operating characteristic (ROC) curve of DSRL and clinical outcome was drawn, the area under ROC curve (AUC) was calculated, and the best truncation value was calculated according to Yoden index. The average value of the best truncation value obtained by the two largest AUCs are taken as the best value of DSRL. IBM SPSS Statistics V26.0 is used for statistical analysis. *p* < 0.05 is considered to be statistically significant.

## 3 Results

### 3.1 Baseline characteristics

From May 2016 to September 2023, a total of 223 children met the inclusion criteria and were included in our analysis, among which 104 cases were short-segment HSCR and 119 cases were long-segment HSCR, 2 cases were complicated by Down syndrome. The median age of the patients was 4 months (IQR 3–6).

#### 3.1.1 Short-segment HSCR

There were 104 children, including 85 males and 19 females. The median age of TEPT was 3.5 months (IQR 3–6). The median total intestinal resection length was 16.1 cm (IQR 14.1–18.4), with a median TZ length of 1.6 cm (IQR 1.0–2.2). Among the three groups, 48 cases had DSRL < 10 cm, 51 cases had 10 ≤ DSRL < 20 cm, and 5 cases had DSRL ≥ 20 cm. Except for preoperative anemia (<10 cm 60.4%,10 ≤ DSRL < 20 cm 72.5%, ≥ 20 cm 20.0%, *p* *=* 0.047), there was no statistical difference in other baseline characteristics among the three groups (Table 1A).

**Table 1A:** Baseline characteristics of children with short-segment HSCR.

Characteristics		All case (*n*)	%	<10 cm(n)	%	10≤ DSRL<20 cm (*n*)	%	≥20 cm (*n*)	%	*p*
Age (months)of Pull through (median, IQR)		3.5 (3–6)		3 (3–5)		4 (3–6)		8 (2.5–19)		0.459
Sex (M/F)	M	85	81.7	36	75.0	45	88.2	4	80.0	0.233
	F	19	18.3	12	25.0	6	11.8	1	20.0	
Gestational age	Full term	96	92.3	44	91.7	47	92.2	5	100.0	0.8
	Premature	8	7.7	4	8.3	4	7.8	0	0.0	
Family history	Y	3	2.9	1	2.1	2	3.9	0	0.0	0.797
	N	101	97.1	47	97.9	49	96.1	5	100.0	
Parity	First child	50	48.1	23	47.9	23	45.1	4	80.0	0.375
	Second child	46	44.2	23	47.9	22	43.1	1	20.0	
	Third child	8	7.7	2	4.2	6	11.8	0	0.0	
Pre Op colitis	Y	16	15.4	9	18.8	7	13.7	0	0.0	0.488
	N	88	84.6	39	81.3	44	86.3	5	100.0	
Pre pull-through ostomy	Y	7	6.7	5	10.4	2	3.9	0	0.0	0.36
	N	97	93.3	43	89.6	49	96.1	5	100.0	
Hypoalbuminemia	Y	68	65.4	28	58.3	37	72.5	3	60.0	0.321
	N	36	34.6	20	41.7	14	27.5	2	40.0	
Anemia	Y	67	64.4	29	60.4	37	72.5	1	20.0	0.047
	N	37	35.6	19	39.6	14	27.5	4	80.0	
Surgical method	Transanal	73	70.2	31	64.6	38	74.5	4	80.0	0.495
	Laparoscope	31	29.8	17	35.4	13	25.5	1	20.0	

#### 3.1.2 Long-segment HSCR

There were 119 children, including 104 males and 15 females. The median age of TEPT was 4 months (IQR 3–6). The median total intestinal resection length was 35 cm (IQR 31–38), with a median TZ length of 3 cm (IQR 1.5–5). Among the three groups, 22 cases had DSRL < 10 cm, 60 cases had 10 ≤ DSRL < 20 cm, and 37 cases had DSRL ≥ 20 cm. There was no statistical difference in baseline characteristics among the three groups (Table 1B).

**Table 1B:** Baseline characteristics of children with long-segment HSCR.

Characteristics		All case (*n*)	%	<10 cm (*n*)	%	10≤ DSRL<20 cm (*n*)	%	≥20 cm (*n*)	%	*p*
Age(months)of pull through (median, IQR)		4 (3–6)		3 (2–5.3)		4 (3–5)		4 (3–9)		0.196
Sex (M/F)	M	104	87.4	21	95.5	52	86.7	31	83.8	0.414
	F	15	12.6	1	4.5	8	13.3	6	16.2	
Gestational age	Full term	115	96.6	22	100.0	58	96.7	35	94.6	0.538
	Premature	4	3.4	0	0.0	2	3.3	2	5.4	
Family History	Y	5	4.2	1	4.5	2	3.3	2	5.4	0.882
	N	114	95.8	21	95.5	58	96.7	35	94.6	
Parity	First child	49	41.2	6	27.3	26	43.3	17	45.9	0.527
	Second child	51	42.9	12	54.5	23	38.3	16	43.2	
	Third child	19	16	4	18.2	11	18.3	4	10.8	
Preoperative enterocolitis	Y	24	20.2	4	18.2	13	21.7	7	18.9	0.917
	N	95	79.8	18	81.8	47	78.3	30	81.1	
Pre pull-through ostomy	Y	8	6.7	3	13.6	1	1.7	4	10.8	0.078
	N	111	93.3	19	86.4	59	98.3	33	89.2	
Hypoalbuminemia	Y	66	55.5	13	59.1	30	50.0	23	62.2	0.469
	N	53	44.5	9	40.9	30	50.0	14	37.8	
Anemia	Y	88	73.9	14	63.6	49	81.7	25	67.6	0.146
	N	31	26.1	8	36.4	11	18.3	12	32.4	
Surgical method	Transanal	49	41.2	8	36.4	26	43.3	15	40.5	0.847
	Laparoscope	70	58.8	14	63.6	34	56.7	22	59.5	

### 3.2 Postoperative clinical outcomes of HSCR

#### 3.2.1 Short-segment HSCR

Three patients with short-segment HSCR underwent reoperations [2 due to retained TZ treated with secondary TEPT, DSRL 3 cm and 15.5 cm (age: 25 months); 1 for anastomotic stenosis treated by anoplasty]. No significant association was found between DSRL and clinical outcomes at 6-month follow-up, as presented in Table 2A. The overall rate of excellent bowel function following surgery was 86.5%, with the DSRL group exhibiting the highest rate of 87.5% for patients with a segment length of less than 10 cm, as indicated in Table 3.

**Table 2A:** Postoperative clinical outcomes in short-segment HSCR.

Characteristics		All case (*n*)	%	<10 cm (*n*)	%	10 ≤ DSRL<20 cm (*n*)	%	≥20 cm (*n*)	%	*p*	The total length of intestinal resection	*p*	The length of aganglionosis	*p*
Postoperative Enterocolitis	Y	34	32.7	19	39.6	14	27.5	1	20.0	0.361	15.8 (14–17.3)	0.139	4 (3–5.6)	0.515
	N	70	67.3	29	60.4	37	72.5	4	80.0		16.5 (15–19.5)		3.6 (3–5.1)	
Constipation	Y	1	1.0	0	0.0	1	2.0	0	0.0	0.592	20.6	0.285	3.2	0.676
	N	103	99.0	48	100.0	50	98.0	5	100.0		16 (14–18.2)		3.8 (3–5.5)	
Reoperation	Y	3	2.9	1	2.1	1	2.0	1	20.0	0.064	19	0.340	2.5	0.501
	N	101	97.1	47	97.9	50	98.0	4	80.0		16 (14.3–18.2)		3.8 (3–5.3)	
Soiling	Y	14	13.5	6	12.5	7	13.7	1	20.0	0.894	15.3 (12.9–17.6)	0.511	3.7 (2.9–5.6)	0.867
	N	90	86.5	42	87.5	44	86.3	4	80.0		16.5 (15–18.6)		3.8 (3–5.1)	
Perianal erosion	Y	6	5.8	4	8.3	2	3.9	0	0.0	0.577	17.5 (14.5–20.5)	0.922	7.5 (3.8–9.4)	0.066
	N	98	94.2	44	91.7	49	96.1	5	100.0		16.1 (14.1–18.2)		3.6 (3–5)	
Medical therapy	Y	8	7.7	6	12.5	2	3.9	0	0.0	0.223	15 (12.3–18.5)	0.831	5.8 (3.5–7.8)	0.054
	N	96	92.3	42	87.5	49	96.1	5	100.0		16.4 (14.9–18.4)		3.6 (3–5)	

#### 3.2.2 Long-segment HSCR

Four patients with long-segment HSCR underwent reoperations (2 due to retained TZ treated with secondary TEPT, DSRL 12 cm and 15 cm; 1 for adhesive intestinal obstruction treated by adhesiolysis; 1 for anastomotic stenosis treated by anoplasty). The clinical outcome of long-segment HSCR is shown in Table 2B. The overall excellent rate of bowel function was 86.6%, with the highest postoperative excellent rate of 93.3% for 10 ≤ DSRL < 20 cm (Table 3). In long-segment HSCR, there was no significant difference between DSRL and other postoperative complications, except for soiling (*p* = 0.002) and perianal erosion (*p* = 0.026), and the postoperative bowel function was the best when 10 ≤ DSRL < 20 cm.

**Table 2B:** Postoperative clinical outcomes in long-segment HSCR.

Characteristics		All case (*n*)	%	<10 cm (*n*)	%	10 ≤ DSRL<20 cm (*n*)	%	≥20 cm (*n*)	%	*p*	The total length of intestinal resection	*p*	The length of aganglionosis	*p*
Postoperative Enterocolitis	Y	43	36.1	9	40.9	20	33.3	14	37.8	0.791	35 (31–40)	0.590	12 (11–18)	0.344
	N	76	63.9	13	59.1	40	66.7	23	62.2		35 (30.1–38)		12 (11–15.5)	
Constipation	Y	6	5.0	0	0.0	3	5.0	3	8.1	0.388	34 (31.5–35.75)	0.609	11.5 (10.9–14.1)	0.497
	N	113	95.0	22	100.0	57	95.0	34	91.9		35 (30.8–38.5)		12 (11–16)	
Reoperation	Y	4	3.4	0	0.0	3	5.0	1	2.7	0.519	34.5 (32.3–37.5)	0.994	16 (13–17.5)	0.145
	N	115	96.6	22	100.0	57	95.0	36	97.3		35 (30.5–38)		12 (11–15)	
Soiling	Y	16	13.4	8	36.4	4	6.7	4	10.8	0.002	41 (33.5–49.5)	0.008	17.5 (11–30)	0.016
	N	103	86.6	14	63.6	56	93.3	33	89.2		35 (30.5–38)		12 (11–14)	
Perianal erosion	Y	6	5.0	4	18.2	1	1.7	1	2.7	0.026	40 (35.5–50.5)	0.037	20 (14.8–26)	0.038
	N	113	95.0	18	81.8	59	98.3	36	97.3		35 (30.4,38)		12 (11–15)	
Medical therapy	Y	97	81.5	16	72.7	49	81.7	32	86.5	0.420	35 (30.8–38)	0.701	12 (11–15)	0.248
	N	22	18.5	6	27.3	11	18.3	5	13.5		35.5 (31–38.5)		12.5 (11–18.6)	

**Table 3:** Postoperative bowel function score in HSCR.

Bowel function score		Short-segment HSCR				Long-segment HSCR			
		All case (*n*)	<10 cm (n)	10 ≤ DSRL<20 cm (*n*)	≥20 cm (*n*)	All case (*n*)	<10 cm (*n*)	10 ≤ DSRL<20 cm (*n*)	≥20 cm (*n*)
Wingspread score	excellent/very Good	89	42	43	4	97	14	53	30
	Good	1	0	1	0	6	0	3	3
	Fair	5	1	4	0	13	7	3	3
	Poor	9	5	3	1	3	1	1	1
Excellent and good rate %		86.5	87.5	86.3	80.0	86.6	63.6	93.3	89.2

### 3.3 The optimal critical value of the cutting length of the dilated segment

#### 3.3.1 Short-segment HSCR

**Figure 2:**
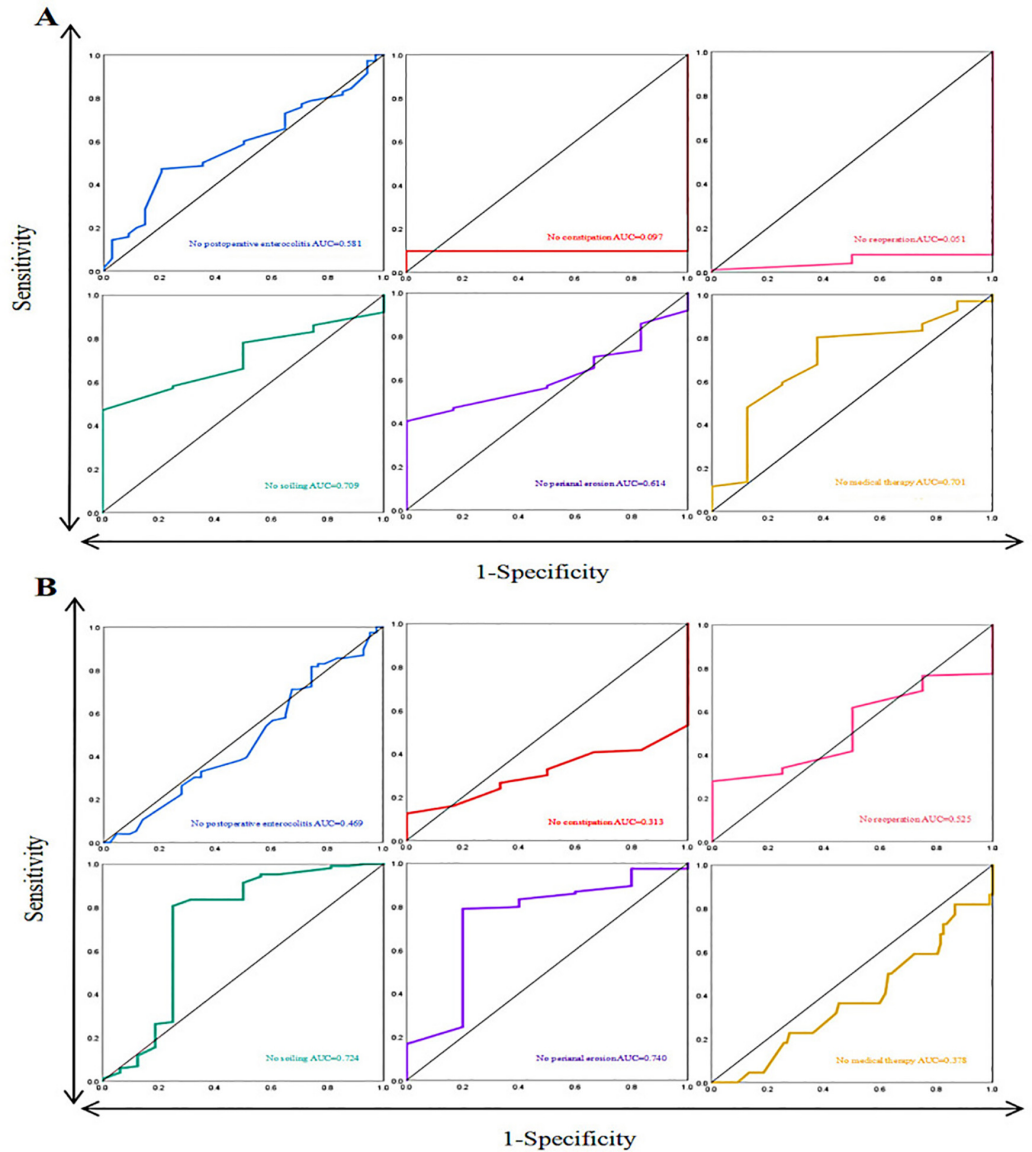
**(A)** ROC curve of short-segment HSCR and clinical outcomes. **(B)** ROC curve of long-segment HSCR and clinical outcomes.

In the ROC curve of short-segment HSCR DSRL and clinical outcome, the largest AUC was no soiling [AUC = 0.709 (*CI*:0.547–0.871)] and no medical therapy [AUC = 0.701 (*CI*:0.515–0.887)] (Figure 2A). Further analysis indicates that DSRL does not have a significant effect on clinical outcomes following short-segment HSCR surgery. However, theoretical calculations suggest that the optimal value, as determined by the Youden index, is 7.25 cm.

#### 3.3.2 Long-segment HSCR

In the ROC curve of long-segment HSCR DSRL and clinical outcome, the largest AUC was no soiling [AUC = 0.724 (*CI*:0.547–0.901)] and no perianal erosion [AUC = 0.740 (*CI*:0.495–0.985)] (Figure 2B). The optimal value calculated using the Youden index is 13.00 cm.

### 3.4 The relationship between the length of aganglionosis and the total length of intestinal resection

**Figure 3:**
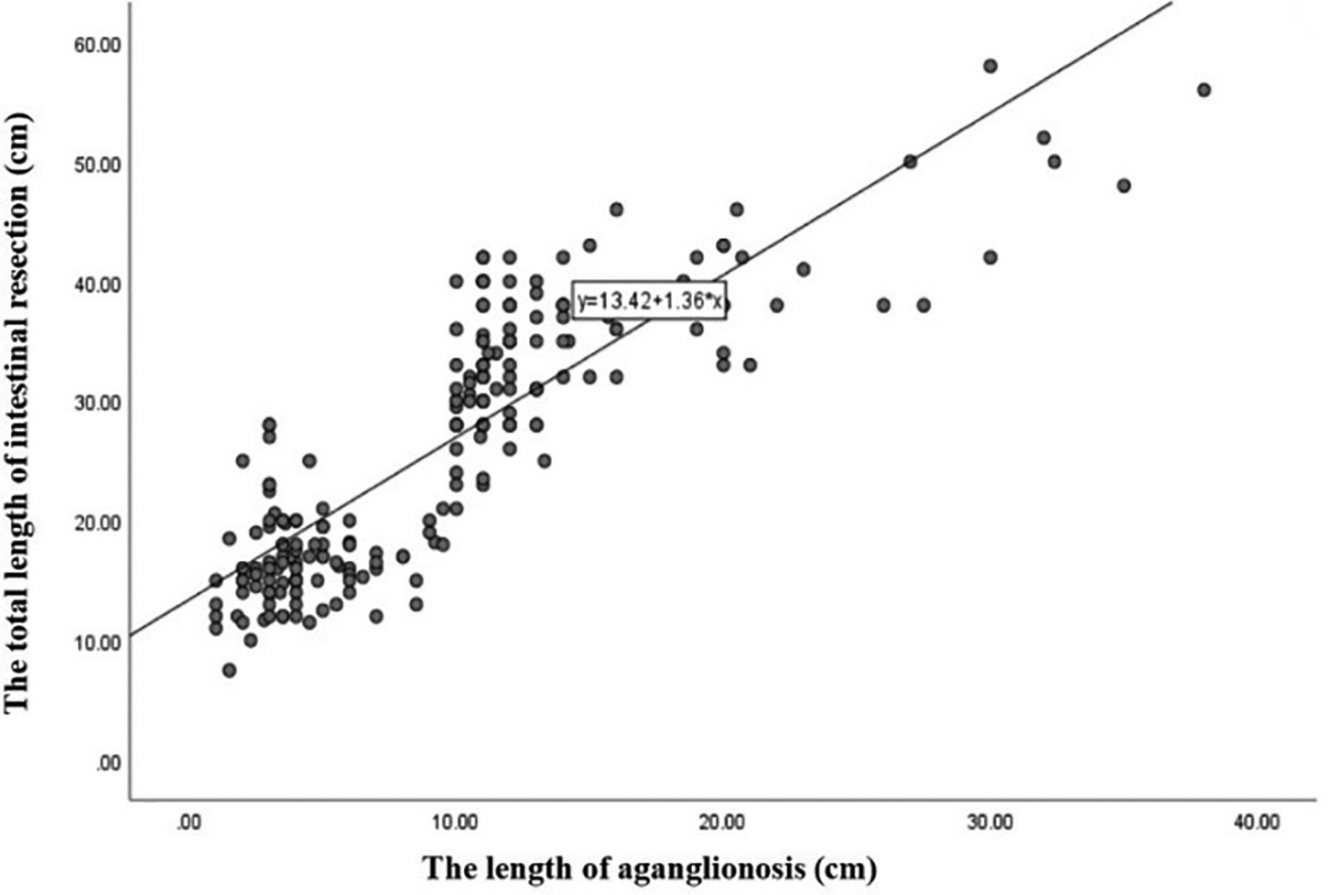
Schematic diagram of the correlation between the total length of intestinal resection and the length of aganglionosis.

A strong positive correlation was observed between the length of the length of aganglionosis and the total length of intestinal resection (Spearman's rs = 0.855, *p* < 0.001) (Figure 3).

### 3.5 The relationship between the total length of intestinal resection, the length of aganglionosis and clinical outcomes in HSCR

In short-segment HSCR, there were no statistically significant difference in the total length of intestinal resection, the length of aganglionosis and postoperative clinical outcomes. However, in long-segment HSCR, the total length of intestinal resection, the length of aganglionosis were associated with soiling (*p* *=* 0.008, *p* = 0.016) and perianal erosion (*p* = 0.037, *p* = 0.038), as detailed in Table 2.

## 4 Discussion

The surgical principle of HSCR is to completely remove the segment of the intestine without ganglion cells and the TZ, and the segment with normal nerve supply should be anastomosed to the anal canal. Currently, there is no standardized guideline regarding the length of the dilated segment that should be resected. Georgeson (14) proposed in his account of laparoscopic-assisted pull-through surgery that the resection should extend 10–15 cm beyond the TZ. Conversely, Schästern et al. (15) advised that the resection should occur approximately 2–3 cm proximal to the first biopsy that indicates a normal density of ganglion cells. Inadequate resection of the dilated segment may result in the presence of a residual TZ, which can adversely influence clinical outcomes. Residual TZ remnants is a significant factor contributing to the necessity for reoperation following HSCR surgery (16). More and more studies have shown that the distribution of TZ is uneven, and White and Langer (17) have found that the “leading edge” of ganglion cells extends up to 2.4 cm in the myenteric plexus and 2.1 cm in the submucosal plexus. Some scholars have even shown that TZ can extend to 13 cm, and there may be a longer TZ in children with long-segment HSCR (5). Conversely, excessive resection of the dilated segment may result in the loss of residual colon, which can compromise water absorption and, in severe instances, hinder growth and development. Therefore, it is imperative to investigate the optimal length of the DSRL and its correlation with clinical outcomes. Supplementary materials are shown in Table 4.

**Table 4:** Literature recommendation DSRL summary table.

Time	Author	Literature	Recommend DSRL (cm)
2002	Georgeson (14)	Laparoscopic-assisted pull-through for Hirschsprung's disease	10–15
2013	Schästern (15)	A practical guide for the diagnosis of primary enteric nervous system disorders	2–3
2019	David Coyle (5)	The Extent of the Transition Zone in Hirschsprung Disease	>5
2020	Kristiina Kyrklund (4)	ERNICA guidelines for the management of rectosigmoid Hirschsprung's disease	At least 5–10
2024	Sarah Ullrich (6)	Does Length of Extended Resection BeyondTransition Zone Change Clinical Outcome for Hirschsprung Pull-Through?	>5

In contrast to previous studies, the present investigation specifically analyzed DSRL across different pathological subtypes of HSCR and explored its association with short-term clinical outcomes. Based on single-center clinical data, we propose optimal DSRL values of 7.25 cm for short-segment HSCR and 13.00 cm for long-segment HSCR, which are associated with favorable prognostic outcomes. These recommendations align with the widely accepted threshold of DSRL > 5 cm suggested by most scholars. Furthermore, our findings demonstrate that DSRL does not significantly impact postoperative clinical outcomes in short-segment HSCR, while it shows a correlation with the incidence of soiling and perianal erosion in long-segment HSCR cases. We anticipate that these findings may provide valuable evidence for personalized surgical planning in clinical practice, potentially optimizing surgical decision-making and improving patient outcomes.

In the course of our follow-up investigation, we found that DSRL was associated with soiling after long-segment HSCR surgery and perianal erosion. A study conducted by Lu et al. indicated that patients with long-segment HSCR exhibited poorer intestinal function compared to those with short-segment HSCR, with a higher propensity for fecal incontinence. This disparity may be attributed to the more extensive colon resection and the diminished water absorption capacity of the colon in long-segment cases (18). Conversely, some researchers argue that there is no significant difference in intestinal function between these two patient groups (19). This discrepancy may stem from the variability in the measurement tools for intestinal function and the surgical techniques employed in prior studies (18). In this investigation, it was observed that 14 cases of children with short-segment HSCR experienced soiling following surgical intervention, while 16 cases of children with long-segment HSCR also reported soiling postoperatively. The incidence of soiling was found to be lowest at 6.7% when 10≤ DSRL<20 cm. A notable strength of this study lies in the fact that all pediatric patients were evaluated using a standardized measurement tool administered by the same healthcare professionals, and all underwent the Soave surgical procedure, thereby minimizing potential biases associated with varying measurement instruments and surgical techniques. Furthermore, the early postoperative period in children with HSCR is characterized by a high frequency of bowel movements, loose stools, and soiling, which may lead to perianal erosion. Nevertheless, it is generally observed that as the functionality of the external anal sphincter improves and new rectal function is established, the muscle layer of the distal rectum compensates for the role of the internal anal sphincter, resulting in a gradual alleviation of symptoms in affected children (20). In this investigation, there were 4 cases with perianal erosion observed postoperatively within the short-segment HSCR cohort, while the long-segment HSCR cohort exhibited 5 instances. Notably, The incidence of perianal erosion was lowest in the long-segment group with 10≤ DSRL < 20 cm, at only 1.7%. This finding aligns with the optimal DSRL value.

Furthermore, the length and extent of the remaining colon warrant particular attention. In clinical practice, short-segment HSCR typically requires rectosigmoid resection, while long-segment HSCR generally necessitates left colon resection (so left hemicolectomy and rectosigmoid resection). For long-segment HSCR cases, the relationship between the length of the remaining colon and postoperative functional recovery is particularly crucial, especially when the residual colon length is relatively short, which warrants closer monitoring of patient prognosis. Our study further revealed that the longer the length of aganglionosis, the greater extent of total bowel resection, corresponding to shorter residual colon length, was associated with increased risks of postoperative soiling and perianal erosion. This phenomenon may be attributed to reduced intestinal storage capacity and accelerated fecal transit resulting from extensive bowel resection, thereby increasing the likelihood of soiling and perianal skin complications. These findings provide a plausible explanation for the higher incidence of soiling and perianal erosion observed in the long-segment HSCR group with DSRL <10 cm.

While this study aimed to investigate the relationship between DSRL and short-term clinical outcomes, our findings ultimately identified the length of aganglionosis as the critical determinant of postoperative prognosis. The clinical findings presented in this study reveal that the DSRL for short-segment HSCR is 7.25 cm, while for long-segment HSCR, it is 13.00 cm. However, there is currently no standardized guideline for determining the optimal DSRL. These measurements are associated with improved clinical outcomes for patients. The decision regarding the appropriate DSRL should be made by taking into account various factors, including the child's age, degree of intestinal dilation and residual colonic function, the condition of the affected bowel, results from preoperative assessments, the surgical technique employed, and the expertise of the surgical team. This process should involve discussions and collaborative decision-making by a multidisciplinary team to establish the most appropriate treatment plan for the child. Furthermore, during the postoperative follow-up, it is crucial to meticulously monitor the child's clinical symptoms, evaluate imaging results, and implement regular biofeedback training, as these measures are vital for enhancing the quality of life and restoring bowel function.

As a retrospective study, this investigation carries inherent limitations, including potential confounding biases such as variations in surgical techniques and subjectivity in pathological assessments. Additionally, sociodemographic factors, including parental cognitive levels, comprehension abilities, and compliance, may have influenced the outcomes. The single-center design, relatively small sample size, and limited follow-up duration may affect the generalizability of our conclusions. It should be specifically noted that while we focused on the impact of DSRL and the length of aganglionosis on outcomes, a comprehensive analysis of morphological characteristics of the dilated segment (e.g., intestinal circumference) was not performed. Although the circumference of the dilated bowel may also be a potential prognostic factor, systematic collection and analysis of these morphological features were limited by the retrospective nature of the data. Future studies should incorporate multicenter, large-scale cohort designs with extended follow-up periods to validate these findings. Furthermore, more detailed subgroup analyses of long-segment HSCR cases are warranted to better evaluate the relationship between residual colon length, morphological characteristics of the dilated segment and clinical outcomes.

## 5 Conclusion

Not only the dilated segment resection length matters for the outcome but also the length of aganglionosis. Clinical data from a single center suggest that the DSRL for short-segment HSCR averages 7.25 cm, while for long-segment HSCR, it is 13.00 cm. These measurements are correlated with improved clinical outcomes for patients. Although DSRL, the total length of intestinal resection and the length of aganglionosis does not significantly influence postoperative clinical outcomes in cases of short-segment HSCR, it is linked to the incidence of soiling and perianal erosion in long-segment HSCR. Nevertheless, the overall quality of life for affected children is reported to be satisfactory.

## Data Availability

The original contributions presented in the study are included in the article/Supplementary Material, further inquiries can be directed to the corresponding author.
